# Mutual information of multiple rhythms in schizophrenia

**DOI:** 10.1007/s00429-023-02744-6

**Published:** 2023-12-13

**Authors:** Antonio Ibáñez-Molina, Yasmina Crespo Cobo, Maria Felipa Soriano Peña, Sergio Iglesias-Parro, Juan Ruiz de Miras

**Affiliations:** 1 Department of Psychology, University of Jaén, Jaén, Spain; 2 Department of Psychology, St. Agustín University Hospital, Av. San Cristóbal, 2D, 23700 Linares, Jaén Spain; 3 Software Engineering Department, University of Granada, Granada, Spain

**Keywords:** Mutual information, EEG rhythms, Frequency coupling, Schizophrenia

## Abstract

Interactions between different cortical rhythms, such as slow and fast oscillations, have been hypothesized to underlie many cognitive functions. In patients diagnosed with schizophrenia, there is some evidence indicating that the interplay between slow and fast oscillations might be impaired or disrupted. In this study, we investigated multiple oscillatory interactions in schizophrenia using a novel approach based on information theory. This method allowed us to investigate interactions from a new perspective, where two or more rhythm interactions could be analyzed at the same time. We calculated the mutual information of multiple rhythms (MIMR) for EEG segments registered in resting state. Following previous studies, we focused on rhythm interactions between theta, alpha, and gamma. The results showed that, in general, MIMR was higher in patients than in controls for alpha–gamma and theta–gamma couplings. This finding of an increased coupling between slow and fast rhythms in schizophrenia may indicate complex interactions in the Default Mode Network (DMN) related to hyperactivation of internally guided cognition.

**Supplementary Information:**

The online version contains supplementary material available at 10.1007/s00429-023-02744-6.

## Introduction

Schizophrenia is a severe disorder characterized by cognitive and social dysfunction. Patients exhibit positive symptoms like hallucinations and delusions, as well as negative symptoms like anhedonia, poor social interactions, and cognitive impairment (Andreasen et al. 1994). Despite ongoing research, the etiology of the disorder remains unclear. However, from the neurophysiological point of view, schizophrenia appears to be a complex disorder involving poor interconnections of large scale neural networks (Dong et al. 2018; Northoff and Duncan 2016). Consequently, the spontaneous activity of the brain could mirror these abnormalities in network function. One of the most widely used methods to investigate the neurophysiology of schizophrenia has been the analysis of the electroencephalograms (EEGs). The majority of studies directed to understanding EEG patterns have used frequency decomposition to obtain measures of power at specific frequency bands. Using this approach, it has been found that schizophrenic patients exhibit a decrease in power within alpha band (8–12 Hz) (Fenton et al. 1980; Kim et al. 2015), and an increase in delta (0–3 Hz) and theta (4–7 Hz) rhythms (Kim et al. 2015). Evidence about faster frequencies is less clear, since some research has found increased power in beta (12–30 Hz) and gamma (> 30 Hz) bands in schizophrenia, while others have found opposite results (for a systematic review, see Maran et al. 2016).

Other approaches to the understanding of schizophrenia through EEGs are designed to detect and analyze connectivity between signals registered from different locations on the scalp of patients. Classically, the main finding has been an increase in coherence widely distributed across the scalp of patients, where coherence can be described as a mathematical method to determine whether two or more sensors, or brain regions, exhibit a similar oscillatory activity. Merrin et al. (1989) extended this finding to unmedicated patients, showing that, at least in theta, there was a high intra- and interhemispheric coherence in this population, compared to controls. Although these findings have been largely replicated (e.g., Kam et al. 2013), reduced coherence has also been described in schizophrenia (Tauscher et al. 1998; Winterer et al. 2001).

More sophisticated methods to obtain measures of synchrony have been proposed to explore connectivity dysfunctions in schizophrenia. For example, Berger et al. (2016), calculated the phase locking value of participants during a working memory task, to assess synchrony between EEG signals in the theta band. They found lower values in frontoparietal connectivity in patients, compared with healthy participants. Similarly, Andreou et al. (2015) reported increased theta imaginary coherence in schizophrenia during resting state. In short, imaginary coherence might be conceived as an update of coherence in which spurious connectivity between signals is prevented (Nolte et al. 2004). In a recent study, Steinman et al. (2020) conducted a review of the most commonly used methods for exploring EEG connectivity in schizophrenia. Although these methods, as we explained above, explore the coherence or synchrony between signals from different recording sources, there are other connectivity measures based on the capacity of one signal to predict the state of a different signal, for example Granger causality (also reviewed in Steinman et al. 2020). Results obtained with these last measures indicated impaired temporoparietal connectivity in the theta frequency band (Kusterman et al. 2018). Considering these findings, it could be posited that there is not a definitive electrophysiological pattern characterizing schizophrenia. The most consistent observation may be an abnormal connectivity within the theta frequency range, both in resting-state and during tasks.

Another approach to understanding the neurophysiological interplay in schizophrenia involves studying cross- frequency coupling between different oscillatory rhythms in the brain. This measure assesses not the interaction among signals at different locations, but the coupling of different frequencies at the same location or cortical source. The coupling of two neural rhythms can be explored using the amplitudes (the magnitude or power) or the phases (the timing) of the signals (Jirsa and Müller 2013). However, one particularly insightful approach has been the study of phase-amplitude coupling (PAC), where the phase of a slow rhythm modulates the amplitude of faster rhythms. This has been demonstrated by Kirihara et al. (2012) and suggests that PAC may reflect a functional mechanism to coordinate activity across distant areas of the cortex (Canolty and Knight 2010). According to this perspective, slow oscillatory activity would coordinate faster oscillations in disparate brain regions. Notably, theta-phase gamma–amplitude coupling has been extensively researched. This specific PAC, where the amplitude of the gamma band is modulated by the phase of the theta band, has been implicated in vital cognitive functions like working memory and long-term memory formation.

In the schizophrenia research, studies have consistently reported reduced theta–gamma coupling during working memory or executive tasks (Barr et al. 2017; Linn and Sponheim 2016; Popov et al. 2015). Furthermore, theta–gamma coupling has been shown to correlate significantly with working memory performance in controls subjects, but not in patients with schizophrenia (Barr et al. 2017). This has led to the hypothesis that dysfunctional theta–gamma coupling may contribute to working memory impairments in schizophrenia. Conversely, theta–gamma coupling appears to be intact or enhanced in patients during resting state (Lee et al. 2020; Won et al. 2018), particularly in prefrontal areas. Hirano et al. (2017) observed increased theta–alpha coupling in patients subjected to passive auditory stimulation. The impaired theta–gamma coupling during cognitive tasks may reflect deficits in higher order cognitive functions in schizophrenia, leading some researchers to propose that the increased resting-state theta–gamma coupling represents a compensatory mechanism for cognitive deficits (Lee et al. 2020; Won et al. 2018). Supporting this hypothesis, Lee et al. (2020) found that increased coupling in patients at rest correlated with better performance in executive tests, providing more evidence to support the idea of a compensatory mechanism that prepares cognitive resources to be available when needed. It is noteworthy that theta–alpha couplings have been also related with cognitive effort and working memory functions (e.g., Rodriguez-Larios et al. 2020). For example, Dimitriadis et al. (2016) found causal couplings between frontal theta and parietal alpha rhythms during mental arithmetic calculations. If both theta–gamma and theta–alpha are involved in effortful cognitive processing at task, they might be part of the same function or closely related functions (for a systematic review on cross-frequency coupling and schizophrenia, see Yakuvov et al. 2022). Moreover, this increased coupling at rest could also be related to recent findings showing a predominance of internally guided cognition in schizophrenia (see Prieto-Alcántara et al. 2020).

A second approach to the study of coupling between different frequency bands in EEG signals draws from Information Theory. This perspective assesses the informational structure of signals to determine levels of randomness within individual signals or to examine information sharing between different signals. Sample Entropy, a common measure of randomness that reflects the unpredictability in a signal's temporal structure, was introduced by Richman and Moorman (2000). This measure was extended to be sensitive to different time scales as a multiscale sample entropy (MSE) (Costa et al. 2002). Although patients with schizophrenia tend to exhibit higher levels of entropy in the EEGs than healthy participants, other studies have shown the opposite pattern of results (for a review, see Fernández et al. 2013). In addition to indicators of single signals, Mutual Information (MI) is one of the preferred measures to explore information sharing between EEG signals. High MI between signals indicates strong linear and nonlinear statistical dependence between signals (high connectivity). Na et al. (2002) explored MI of EEG channels and found an increase in MI for patients with schizophrenia. Carlino et al. (2015) also found a general increase in MI in schizophrenia at temporal, parietal, and occipital regions; moreover, when they compared the increase in MI from eyes-closed to eyes open-conditions, they found that only healthy controls flexibly changed their MI connectivity in their EEGs. In another study, Yin et al. (2017) analyzed the topographical properties of networks constructed with measures of MI between EEG channels and concluded that there is less information sharing in networks from patients than healthy controls. Overall, findings from different authors do not converge into the same pattern of results when information measures are applied, and thus, further experiments are needed to shed more light on the relationship between schizophrenia and coupling of EEG signals. One promising methodological candidate to explore informational aspects of EEGs in schizophrenia would be an exploration of interaction of its informational content at different scales. In the same manner as the above-mentioned PAC measures explore interactions between phase and amplitude of two different frequencies, it would be possible to investigate cross-frequency interactions using mutual information of multiple rhythms (MIMR). This measure was recently developed by Ibáñez-Molina et al. (2020) to explore MI between two or more frequencies in a given signal.

In the present work, we have evaluated MIMR with a particular focus on theta–gamma interactions in patients with schizophrenia and controls at rest. As we mentioned before, previous findings seem to suggest an increased theta–gamma coupling in patients at rest, and we aimed to explore these cross-frequency interactions from the perspective of Information Theory. Since theta–gamma enhancements at rest can be explained with cognitive compensation in patients, in this study, we also investigated if theta–alpha couplings, which are related to cognitive effort, were also increased in patients.

A key advantage of MIMR over other information-theoretic measures is its capacity to elucidate multivariate interactions (as detailed in the methods section). Therefore, our objective was to probe the mutual information across different rhythms in both patients with schizophrenia and healthy controls. We hypothesize that if theta–gamma and theta–alpha couplings are functionally interrelated, as suggested, we would also observe an increase in the theta–alpha–gamma interaction within the patient group.

## Materials and methods

### Participants

The sample of this study was composed of two groups of participants, a group of patients and a group of healthy controls.

The patient group (hereafter SCZ group) was composed of 11 participants recruited at the Mental Health Day Centre at St. Agustín University Hospital (Linares, Jaén). The inclusion criteria were an ICD-10 diagnosis of schizophrenia (F20), psychotic disorder (F23), or schizoaffective disorder (F25). The participants were diagnosed by the clinician in charge of the patient. The mean age in this group was 36.23 years (SD = 10.28 years; min = 23, max = 53). Out of the total participants, 2 (18%) were women. All participants were right-handed. Regarding educational level, 2 (18%) had primary education, 8 (72%) had secondary education, and 1 (10%) had higher education. The mean duration of the disorder (defined as the number of years since diagnosis) was 15.72 years (SD = 10.19 years; min = 3, max = 35). All participants were receiving atypical antipsychotics. Out of the total number of participants, 2 were receiving oral medication and 9 were receiving it in injectable form. In addition, one participant was receiving antidepressants. Due to the disparity of active principle, doses and administration formats, we converted all antipsychotic doses to chlorpromazine equivalents (*M = *818.18 mg, SD = 407.75 mg).

The 20 participants in the control group (hereafter referred to as the Ctrl group) were recruited from among the students of the University of Jaen, from an adult school in Jaen and from the staff of St. Agustín University Hospital (Linares, Jaén). The mean age of this group was 40.72 years (SD = 11.96 years; min = 23, max = 57). Of the total number of participants, 7 (35%) were women; and 18 (90%) were right-handed. Regarding educational level, 1 (5%) had primary education, 12 (60%) had secondary education, and 7 (35%) had higher education. No significant differences were found between groups in terms of educational level (*χ*^2^(2) = 3.29; *p* = 0.19), or gender (*χ*^2^(2) = 0.97; *p* = 0.32). Since age in the Control group did not follow a normal distribution (Shapiro–Wilk = 0.89; *p < *0.05), we used the Mann–Whitney test to compare groups. The results indicated that there were no significant differences in age between the groups (*U* = 85; *p* = 0.31).

For both groups, the exclusion criteria were: a concurrent diagnosis of a neurological disorder, a concurrent diagnosis of a substance abuse disorder, a history of developmental disability, and an inability to sign informed consent. Additionally, a criterion for exclusion in the control group was a diagnosis of a mental disorder (as reported verbally by the participants). All participants provided written informed consent in accordance with the Declaration of Helsinki, and the Jaén Research Ethics Committee approved the study.

### Procedure and data recording

The study was conducted in a hospital laboratory room, enabled for EEG recording. This room had an approximate size of 15 m^2^ and was located in a quiet place with few potentially interfering electrical fields in the band of 50 Hz. Since patients came to the Mental Health Unit in the morning, the EEG recordings of all study participants were made only during that period of time. Participants who agreed to participate were scheduled in the EEG recording lab individually, where the objective of the study was explained to them, the experimental protocol was described and, if they chose to participate, they were requested to sign the informed consent form. The experimenter proceeded to place the 31 active electrode assembly on the 10–20 system with positions FP1, FP2, F7, F3, Fz, F4, F8, FT9, FC5, FC1, FC2, FC6, FT10, T7, C3, C4, T8, TP9, CP5, CP1, CP2, CP6, TP10, P7, P3, Pz, P4, P8, O1, Oz, and O2. We used Cz as the physical reference electrode. Impedances were kept below 5 kOhm. Measurements were carried out using a 62-channel BrainAmp system. Signals were recorded at a frequency of 500 Hz.

Participants were situated in a comfortable chair with a laptop positioned on a desk directly before them, the screen being approximately 70 cm from their eyes. During the resting-state task, they were directed to fixate on a light grey cross at the center of a black background on the laptop screen for a duration of 5 min. Participants were instructed to remain still, refrain from speaking, and were permitted to let their thoughts wander freely. The experimenter monitored the session from behind the participants, ensuring that they remained out of the participants' sight, within the same room.

### EEG processing

Data processing was performed with EEGLAB (Delorme and Makeig 2004) and custom MATLAB functions. For each participant, we selected 5 min continuous data. Blinks and other artifacts were extracted using infomax ICA (Bell and Sejnowski 1995). ICA components with artifacts were selected by visual inspection of the scalp topography, power spectra, and raw activity from all components. The resulting EEGs after denoising were used as inputs for a custom MATLAB script developed to obtain MIMR at the chosen frequencies.

### Mutual information of multiscale rhythms (MIMR)

To obtain the MIMR, we computed binary sequences corresponding to the desired timescales from the original signal. To calculate these binary sequences, we used smoothed versions of the original signal as thresholds. These smoothed versions were obtained by applying median moving windows of different length to the original signal.

Specifically, we initially filtered the original signal using different moving window sizes, where wider windows produced lower frequency signals, while shorter windows resulted in higher frequency signals. Next, to obtain a binary sequence at a given scale, we subtracted the data points of two smoothed versions using successive window sizes, assigning a 1 if the difference of the subtraction was positive and 0 otherwise. Hence, the resulting binary sequence would reflect the rapid activity that is not present in the smoother version obtained with a shorter window. To relate window size to a specific frequency, it is sufficient to know the sampling frequency of the signal. For instance, for a sampling frequency of 1000 Hz, a window size of 201 points would correspond to a frequency of 1000/201 ~ 5 Hz and a window size of 101 points would correspond to a frequency of 1000/101 ~ 10 Hz. Note that this rule provides an approximate window size that captures the maximum wavelength present in the signal (low-pass filter). It is the comparison of this signal with another one, smoothed with a shorter window, which gives the binary sequence at a particular rhythm or frequency band.

For example, if the original series is smoothed using a 201-point window, and these values are compared to another smoothed version with a 101-point window, the binary sequence obtained using the difference would reflect the differential activity between the two scales. In this particular example, the binary sequence obtained with the subtraction of both smoothed signals would contain the activity in the 5 and 10 Hz range.

**Fig. 1:**
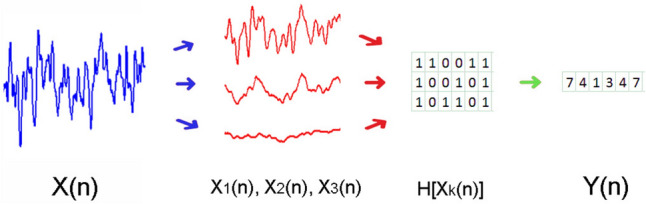
Graphical representation showing the steps to obtain the symbolic series *Y*(*n*) necessary for MIMR calculation. The first step is to obtain smoothed versions of the original signal and use them as thresholds to produce binary series *H*[*X*_*k*_(*n*)]. Each column of this resulting binary matrix is considered a state of the system. In the example of the figure, the first column would be [1 1 1] which in a base 10 would be 7

After calculating all the binary frequencies of interest, they were transformed into a single signal. This new signal was a sequence composed, at each time step, of integers, each of which symbolically represented the binary values at each scale as a single integer in base 2. Finally, for simplicity, these binary numbers were converted into base-10 integers (see Fig. 1 for a graphical description of the whole procedure). For example, if we obtained three binary sequences, and at time step *t*, the values of each were 1, 0, and 1, then we took the number 101 as a base 2 number, and transformed it to 5 in base 10. Note that in this case, 5 represents a state with specific information about the three rhythms obtained with each binary sequence.

From the integers in this symbolic series {*Y*(*n*)}, we obtained the MIMR as the delayed mutual information

1 $${\text{MIMR}} = I\left( {Y(n - \tau ),Y\left( n \right)} \right),$$

where the parameter *τ* can be estimated from the autocorrelation function of *Y*(*n*), and it was set to *τ* = *10*. This measure provides the average number of predicted bits in *Y*(*n*) given the state *Y*(*n *− τ). It is a way to calculate to what extend a given state *Y*(*n*) of the signal would be predicted by the past state *Y*(*n *− *τ*). It evaluates, therefore, the linear and nonlinear temporal dependencies within a time series, and it has been used to quantify the linear and nonlinear statistical coupling between biomedical signals (Escudero et al. 2009). For a more detailed and formal description of the measure, see Ibáñez-Molina et al. (2020).

In the process of MIMR calculation for this study, we used different window sizes (WS: 14, 50, 100) to obtain the binary sequences necessary for *Y*(*n*). These window sizes were selected to approximately capture classical rhythms of θ (~ 5 Hz), α (~ 10 Hz), *γ* (~ 35 Hz), respectively. Given that the sampling rate of the EEG signal was 500 Hz, 500/14 would give an approximation of the 35 Hz wavelength. The same rationale could be applied to 50 and 100 window sizes.

### Comparison metrics

With the aim to better interpret or validate the results obtained with MIMR, we included Sample Entropy and PAC analyses in theta–gamma and theta–alpha couplings.

Sample Entropy assesses the EEG signals from an informational perspective without considering explicit rhythmic interactions. Given that MIMR is also information-based, comparing it with Sample Entropy—a measure that captures statistical dependencies across all scales—provides insight into potential cross-frequency interactions at various scales.

PAC examines cross-frequency interactions through the lens of phase–amplitude interactions. A correspondence in the pattern of results between MIMR and PAC might indicate that MIMR captures similar physiological mechanisms as those involved in PAC. We conducted analyses on both control participants and patients across all electrode sites, applying the Modulation Index method as outlined by Tort et al. (2010). We generated three band-passed signal versions for each relevant frequency band using a zero-phase Finite Impulse Response (FIR) filter in EEGLAB: 33–37 Hz for the gamma band, 8–12 Hz for the alpha band, and 3–6 Hz for the theta band. The gamma band's amplitude and the phases of the alpha and theta bands were extracted using the Hilbert transform. Subsequently, to examine the influence of slower frequencies on the power of the gamma band, we computed the Modulation Index for both theta–gamma and alpha–gamma couplings.

### Data analysis and results

To investigate the coupling between different frequency bands in patients and controls, we calculated MIMR for different combinations of window sizes (WS:14–50, WS:50–100, WS:14–100 and WS:14–50-100), assuming that we were mapping different combinations of frequencies (α-γ, θ-α, γ-θ, and α-θ-γ, respectively).

To examine the distribution of these differences between patients and controls in the cortical topology, we performed comparisons at the sensor level. Since, in most of the sensors the MIMR distribution was not normal, we conducted group comparisons using the Wilcoxon signed rank test. Due to the issue of multiple testing errors, p values of comparisons were corrected by calculating the false discovery rate using the Benjamini and Hochberg’s (1995) method. The analyses were performed with R version 4.0.3 (2020). All figures shown in this study were constructed with violin box-plots. These plots consisted of boxes delineating quartile information, with Q1 being the lower side, Q2 as the median represented as the central line, and Q3 the upper side. The length of the line represent the range of the data, and the colored curves illustrate the probability distributions.

### WS:14–50 (alpha–gamma coupling)

**Fig. 2:**
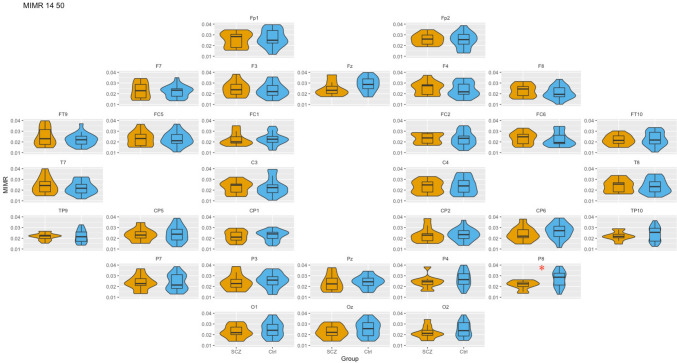
Box-plot of MIMR obtained for 14–50 windows’ size (*α *− *γ* interaction) in each sensor for SCZ and Ctrl groups. Only significant differences are highlighted. For each test, a false discovery rate (FDR) correction was applied to correct for multiple comparisons and minimize false positives

First, we examined the coupling between a fast and a slow rhythm across the topological map in patients and healthy controls. The results of the comparisons between SCZ and Ctrl groups in each of the channels are represented graphically in Fig. 2. Quantitative information on the value of the *U* statistic and the *p* value associated can be found in Supplementary Material.

As can be seen, there were hardly any differences between groups in MIMR, except in P8, where alpha–gamma coupling was significantly higher in controls than in patients.

### WS: 50–100 (theta–alpha coupling)

**Fig. 3:**
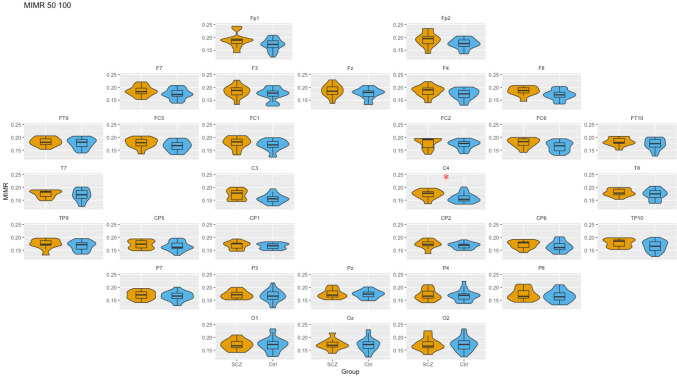
Box-plot of MIMR obtained for 50–100 windows size (*θ* − *α* interaction) in each of the sensors for SCZ and Ctrl groups. Only significant differences are highlighted. For each test, a false discovery rate (FDR) correction was applied to correct for multiple comparisons and minimize false positives; * represents *p < *0.05

Second, we analyzed the coupling between two slow frequencies, alpha and theta (see Fig. 3). Detailed results of these comparisons can be found in the Supplementary Material.

As can be seen, we found significant differences between groups; theta–alpha coupling was greater in patients than in controls, in the right frontal–central region. Although not significant, this same trend was observed in many sensors, as well as the reversed effect at P8 that had been previously observed for alpha–gamma coupling.

### WS: 14–100 (theta–gamma coupling)

**Fig. 4:**
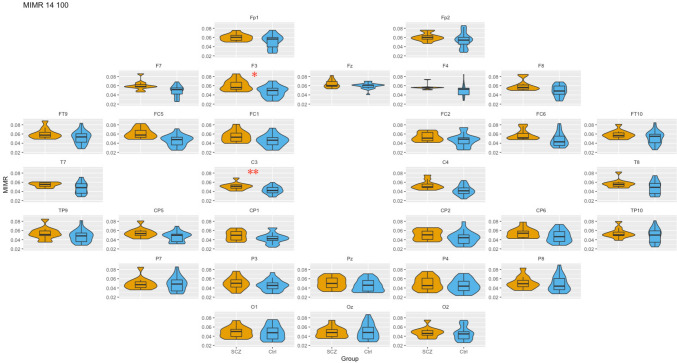
Box-plot of MIMR obtained for 14–100 windows size (*θ* − *γ* interaction) in each of the sensors for SCZ and Ctrl groups. Only significant differences are highlighted. For each test, a false discovery rate (FDR) correction was applied to correct for multiple comparisons and minimize false positives; * represents *p < *0.05, ** represents *p < *0.01

Third, we compared the coupling between two extreme frequencies, one slow (theta) and one fast (gamma). The results are summarized graphically in Fig. 4 (for more detailed information see Supplementary Material).

As can be seen in Fig. 4, the differences now appear bilateralized and with a frontal–central location. In addition, it is worth mentioning the high values but with reduced variability of MIMR in patients in the prefrontal region.

### WS:14–50-100 (theta–alpha–gamma coupling)

**Fig. 5:**
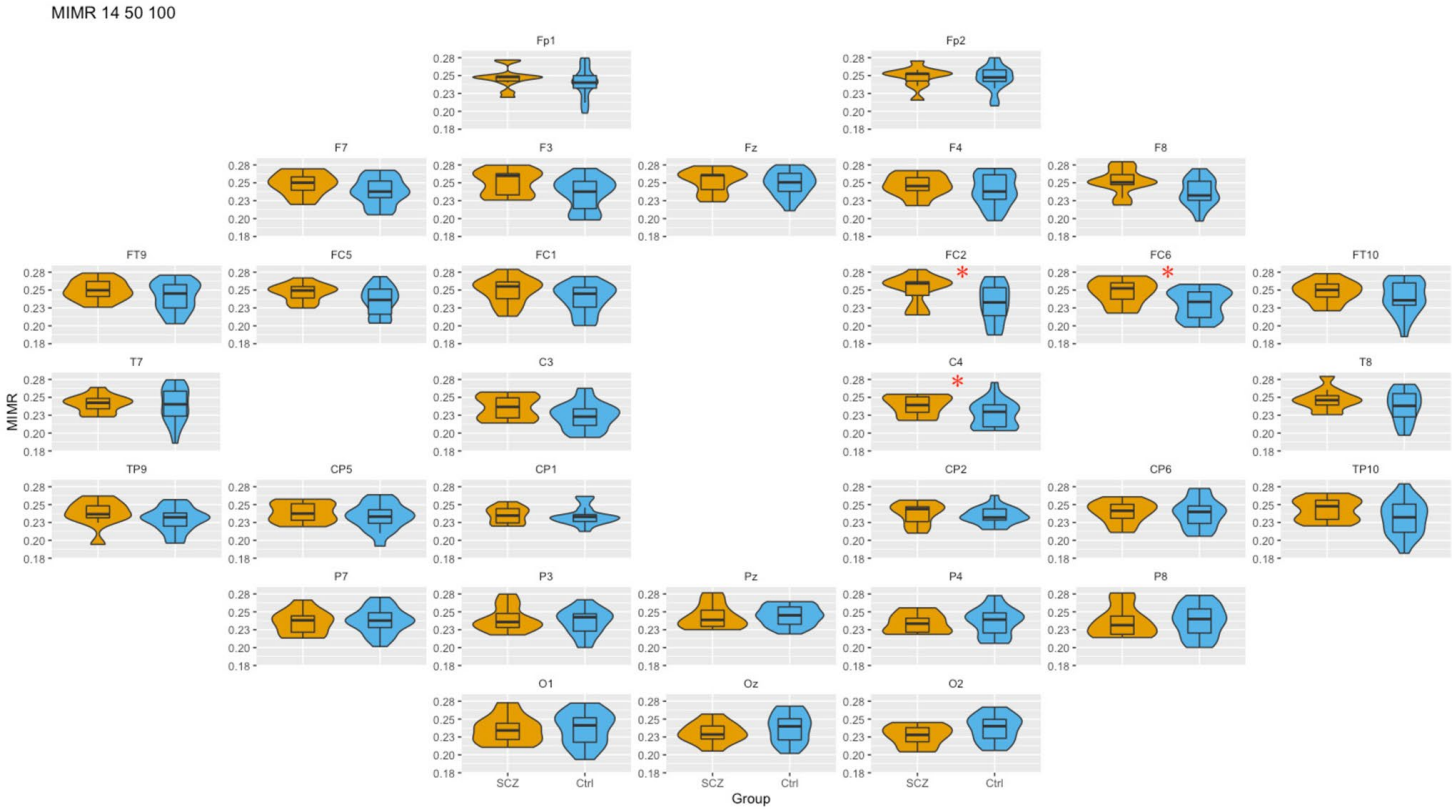
Box-plot of MIMR obtained for 14–50-100 windows size (θ–α–γ interaction) in each of the sensors for SCZ and Ctrl groups. Only significant differences are highlighted. For each test, a false discovery rate (FDR) correction was applied to correct for multiple comparisons and minimize false positives; * represents *p < *0.05

So far, we have studied how the coupling between pairs of frequency bands differed between patients and controls along the scalp topology. In this section, we analyzed the coupling among three frequency bands (*θ*, *α,* and *γ*). Similar to the previous sections, additional detailed information can be found in the Supplementary Materials. A graphical summary of these comparisons is presented in Fig. 5.

In this case, we found significant differences between groups only in the right hemisphere.

### Comparison metrics

To further explore the informational and coupling characteristics of the signals, we also conducted the Sample Entropy and theta–gamma and alpha–gamma PAC measures.

### Sample entropy

As stated in the previous section, Sample Entropy was calculated to discriminate between patients and controls through scalp topology. None of the differences were significant. Detailed results of the comparisons can be found in Supplementary Material.

### PAC measures

As indicated above, we calculated the Modulation Index as a PAC measure for theta–gamma and alpha–gamma. We applied the same statistical analysis to those in MIMR showing that there were no significant differences between control and patients at any electrode site (See Supplementary Material for details).

## General discussion

In this study, we investigated couplings between different frequency rhythms at various electrode sites in patients with schizophrenia and healthy controls using MIMR. We specifically examined theta–gamma, theta–alpha, alpha–gamma, and, uniquely, theta–alpha–gamma couplings—the latter of which has not been previously studied, as past research has focused solely on cross-frequency coupling between two rhythms. MIMR analysis discerns discrete states by converting the amplitude of filtered signals at specific frequencies into binary form, defining a state at any given moment. It captures the statistical dependence in these binary series, thus being sensitive to the recurrence of state patterns.

Our findings revealed an increased bilateral theta–gamma coupling in patients, particularly in frontal and central regions, aligning with prior research that indicates heightened theta–gamma coupling in resting patients. Studies by Lee et al. (2020) and Won et al. (2018) have observed that theta–gamma coupling remains preserved or increased in resting patients, notably in prefrontal areas. Conversely, a reduction in theta–gamma coupling has been noted during cognitive tasks. Barr et al. (2017) reported impaired theta–gamma coupling during an N-Back task that adjusted working memory load. Similarly, Grove et al. (2021) identified decreased theta–gamma coupling between posterior and anterior sites in schizophrenia patients during a gaze detection task. Collectively, these findings suggest a pattern of compromised theta–gamma coupling during tasks, yet an elevated coupling at rest. This increased resting coupling may serve to make cognitive resources readily available to patients, possibly as a preparatory mechanism for upcoming cognitive demands (Lee et al. 2020).

At right frontal and central sites, theta–alpha coupling was also higher in patients than in healthy participants. In the case of theta–alpha coupling, it has been also associated with cognitive effort and working memory function (Dimitriadis et al. 2016; Kawasaki et al. 2010). Recently, in a study by Rodriguez-Larios et al. (2020), it was reported a monotonically increasing theta–alpha coupling during meditation, rest, and an arithmetic operation task, suggesting a reinforcement of the interaction between memory and executive functions as the level of cognitive demands increases. Our finding of increased theta–alpha coupling at rest could be interpreted in the same line as theta–gamma coupling, as reflecting a compensatory mechanism triggered during rest to prepare the cognitive system for an eventual effort. Another possibility would be that this increased coupling is reflecting a hyperactivation of the DMN in patients at rest. Consistent with this idea, Guo et al. (2017), in an fMRI experiment, found that drug-naive patients showed more activation in prefrontal regions of the DMN than patients’ relatives and healthy controls. In addition, medial parietal regions were more activated in patients and relatives when compared with healthy controls. Similar results were reported by Whitfield-Gabriely et al. (2009), who found that participants and first-degree relatives exhibited less DMN deactivation than healthy participants in the transition between resting state and a working memory task. Therefore, our results, which show an increase in MIMR for theta–gamma and theta–alpha rhythms, are consistent with the hypothesis that, during rest, the DMN or specific subsystems of the DMN are activated to a greater degree. Moreover, in an interesting review paper, Hu et al. (2017) reported that the majority of studies about DMN in schizophrenia have shown an increase in functional connectivity when compared with healthy controls, and reinforces the claiming that DMN hyperactivity is an important characteristic of schizophrenia that could be related with measures of EEG coupling.

It is noteworthy that, when we calculated the coupling across theta–alpha–gamma, an increase of MIMR was found at right frontal–central electrodes. This three-way interaction reinforces the hypothesis that the previously described results come from the same neural mechanism. It would be possible that the increased theta–gamma, theta–alpha, and theta–alpha–gamma couplings reflected the activity of complex interactions between DMN regions. This interaction could be, as we mentioned before, compensatory; that is, an effort to anticipate a task demand (in fact, both theta–gamma and theta–alpha are mostly related with processes of working memory). However, another possibility is that increased coupling at rest is reflecting hyperactivation of networks related to internally guided cognition, such as autobiographical memory or mind wandering. This hypothesis is supported by recent findings of a greater frequency of mind wandering in patients with schizophrenia (Iglesias-Parro et al. 2020).

Finally, we have also analyzed alpha–gamma coupling, where we found increased alpha–gamma coupling in controls only at the P8 electrode. Although we cannot give a particular meaning to the spatial location of this effect, it is consistent with previous research showing that patients diagnosed with schizophrenia exhibited low alpha–gamma interaction during a task of sensory information processing (White et al. 2010). In addition, there are specific studies that showed a reduced alpha–gamma coupling in prefrontal areas in patients (Davoudi et al. 2021; Murphy et al. 2020; Yakubov et al. 2022). In general, this coupling deficit in patients with schizophrenia has been related to the interruption of the prioritization of visual representations in working memory (Davoudi et al. 2021).

In the study, we present here we use a measure based on information theory. The motivation for the use of this approach was to gain new insight in coupling across frequency bands through a measure with no a priori assumptions. Previous measures of cross-frequency coupling cannot capture all coupling manifestations (Cohen 2008). For example, the oscillation components need to be prominent or well defined to capture interactions between rhythms. In addition, these measures are sensitive to a specific interaction between frequencies. The PAC measure, for example, is only sensitive to phase–amplitude interactions from slow and fast frequencies. In fact, the analyses on our data did not find any effect of gamma–amplitude modulation by slower rhythms, suggesting that the nature of coupling interactions were not only restricted to amplitude–phase modulations. On the contrary, MIMR can be sensitive to many types of interactions, including n-rhythm coupling. In this study, we show that there is a three-way interaction between rhythms resembling theta–alpha–gamma, which is congruent with the existence of a single mechanism underlying increased coupling in schizophrenia during resting state. In addition, the fact that sample entropy failed to show differences between patients and controls indicates that the locus of the divergence between those groups was the coordination between cortical activities at different scales. However, the sample size for the patients was relatively small compared to healthy participants, and that could be the reason for the lack of statistical power in PAC and Sample Entropy.

### Supplementary Information

Below is the link to the electronic supplementary material.

Supplementary file1 (DOCX 25 kb)

## Data Availability

Enquiries about data availability should be directed to the authors.
