# Supplementary material for: Mutual information of multiple rhythms in schizophrenia
Source: Brain Struct Funct. 2023 Dec 13;229(2):285–95. doi: 10.1007/s00429-023-02744-6 (PMC10917874; doi:10.1007/s00429-023-02744-6)
Supplement: Supplementary file 1 — Supplementary file1 (DOCX 25 kb) [file 429_2023_2744_MOESM1_ESM.docx]

**Supplementary Material**

**MIMR calculation**

MIMR is a delayed mutual information of the Y(n) time series:

$$MIMR = I(Y(n-\tau),Y(n))=\sum_{n} P(Y(n-\tau),Y(n)) {log}_{2}\frac{P(Y(n-\tau),Y(n))}{P(Y(n-\tau)) P(Y(n))}$$

The vector signal Y(n) comes from the binary matrix $H_{k}\left[ x_{k}\left( n \right) \right].$ To obtain this binary matrix, we first construct k versions of the original signal x(n). Each version is a median based smoothed signal with a specific window size. Each row of $H_{k}\left[ x_{k}\left( n \right) \right]$ is the result of applying the Heaviside function to the subtraction of two consecutive versions of the signal. In the case of k=1 we use the subtraction of the original signal and the smoothed version with the smaller window size. Note that this smoothing procedure could be replaced by low pass filtering. Finally, we take the values of each column of $H_{k}\left[ x_{k}\left( n \right) \right]$ and considered a single number in base 2 which we simply transform in a base of 10 for Y(n). In addition, we selected a value of the delay parameter $\tau$ at the approximate mean time across participants and conditions at which we observed the first drop in the autocorrelation function of the EEG series.

For example, in this study we used three smoothed versions of the signal with window sizes of 14, 50 and 100. The first row of $H_{k}\left[ x_{k}\left( n \right) \right]$ would be the Heaviside function of x(n) – x_1_(n) where x(n) is the original signal and x_1_(n) is a smoothed version using a window size of 14; the second row would be based on x_1_(n)-x_2_(n) where x_2_(n) is a smoothed version of x(n) using a window size of 50, and so on. In this specific case, to obtain Y(1) we need to take the values of $H_{k}\left[ x_{k}\left( 1 \right) \right]$. If they were for example {1,0,1} we consider this values as the number 101 which in base of 10 is 5.

**Table 1**

Wilcoxon signed rank test statistic (*U*) and significance (*p*-value) corrected for each sensor in each combination of window sizes (WS) studied. We also include the statistical significance between groups of PAC for theta-gamma and alpha-gammacouplings

|  | WS 14-50 | | WS 50-100 | | WS 14-100 | | WS 14-50-100 | | Samp Entropy | | Theta-gamma | | Gamma-Alpha | |
| --- | --- | --- | --- | --- | --- | --- | --- | --- | --- | --- | --- | --- | --- | --- |
| Channel | *U* | *p*-value | *U* | *p*-value | *U* | *p*-value | *U* | *p*-value | *U* | *p*-value | *U* | *p-value* | *U* | *p-value* |
| **Fp1** | 109 | 0984 | 148 | 0122 | 146 | 0143 | 124 | 0577 | 90 | 0421 | 202 | 0586 | 227 | 0337 |
| **Fp2** | 117 | 0788 | 152 | 0087 | 148 | 0121 | 105 | 0853 | 83 | 0274 | 215 | 0454 | 212 | 0485 |
| **F7** | 126 | 0522 | 145 | 0154 | 165 | 0024 | 145 | 0154 | 100 | 0695 | 221 | 0395 | 204 | 0566 |
| **F3** | 112 | 0951 | 140 | 0223 | 158 | 005 | 135 | 0312 | 82 | 0256 | 147 | 0947 | 202 | 0586 |
| **Fz** | 113 | 0918 | 139 | 0239 | 124 | 0576 | 115 | 0853 | 90 | 0421 | 194 | 0663 | 186 | 0734 |
| **F4** | 111 | 0984 | 148 | 0122 | 158 | 005 | 154 | 0073 | 75 | 0154 | 220 | 0404 | 206 | 0546 |
| **F8** | 117 | 0788 | 157 | 0055 | 155 | 0066 | 153 | 0079 | 69 | 0095 | 250 | 0157 | 244 | 0197 |
| **FT9** | 122 | 0635 | 129 | 0445 | 145 | 0154 | 137 | 0274 | 82 | 0256 | 126 | 0984 | 149 | 0941 |
| **FC5** | 115 | 0853 | 147 | 0132 | 184 | 0002 | 144 | 0167 | 90 | 0421 | 205 | 0556 | 197 | 0635 |
| **FC1** | 100 | 0695 | 134 | 0332 | 148 | 0122 | 139 | 0239 | 86 | 0332 | 245 | 019 | 207 | 0536 |
| **FC2** | 109 | 0984 | 140 | 0223 | 158 | 005 | 158 | 005 | 84 | 0292 | 193 | 0672 | 151 | 0935 |
| **FC6** | 115 | 0853 | 161 | 0037 | 166 | 0022 | 163 | 003 | 75 | 0154 | 271 | 0062 | 226 | 0346 |
| **FT10** | 100 | 0695 | 143 | 018 | 139 | 0239 | 129 | 0445 | 74 | 0143 | 193 | 0672 | 202 | 0586 |
| **T7** | 111 | 0984 | 121 | 0665 | 159 | 0045 | 115 | 0853 | 85 | 0312 | 125 | 0985 | 119 | 099 |
| **C3** | 117 | 0788 | 153 | 0079 | 173 | 001 | 153 | 0079 | 86 | 0332 | 214 | 0464 | 201 | 0596 |
| **C4** | 108 | 0951 | 163 | 003 | 178 | 0005 | 161 | 0037 | 84 | 0292 | 197 | 0635 | 240 | 0226 |
| **T8** | 107 | 0918 | 134 | 0332 | 146 | 0143 | 127 | 0496 | 85 | 0312 | 202 | 0586 | 167 | 0866 |
| **TP9** | 103 | 0788 | 127 | 0496 | 141 | 0208 | 143 | 018 | 96 | 0577 | 238 | 0242 | 213 | 0475 |
| **CP5** | 104 | 082 | 131 | 0397 | 154 | 0073 | 123 | 0606 | 85 | 0312 | 165 | 0877 | 165 | 0877 |
| **CP1** | 95 | 0549 | 130 | 0421 | 136 | 0292 | 121 | 0665 | 96 | 0577 | 133 | 0976 | 124 | 0986 |
| **CP2** | 92 | 047 | 135 | 0311 | 147 | 0132 | 133 | 0352 | 98 | 0635 | 179 | 0789 | 214 | 0464 |
| **CP6** | 815 | 0248 | 141 | 0208 | 142 | 0193 | 121 | 0665 | 93 | 0496 | 225 | 0356 | 190 | 0699 |
| **TP10** | 92 | 047 | 153 | 0079 | 129 | 0445 | 140 | 0223 | 85 | 0312 | 157 | 0914 | 126 | 0984 |
| **P7** | 96 | 0577 | 127 | 0496 | 110 | 1 | 105 | 0853 | 85 | 0312 | 188 | 0717 | 203 | 0576 |
| **P3** | 80 | 0223 | 124 | 0577 | 134 | 0332 | 110 | 1 | 100 | 0695 | 164 | 0882 | 182 | 0766 |
| **Pz** | 795 | 0215 | 110 | 1 | 141 | 0208 | 95 | 0549 | 97 | 0606 | 201 | 0596 | 265 | 0082 |
| **P4** | 83 | 0274 | 115 | 0853 | 134 | 0332 | 110 | 1 | 114 | 0885 | 136 | 0971 | 127 | 0983 |
| **P8** | 58 | 0033 | 128 | 047 | 128 | 047 | 118 | 0757 | 103 | 0788 | 238 | 0242 | 225 | 0356 |
| **O1** | 82 | 0256 | 112 | 0951 | 114 | 0885 | 91 | 0445 | 109 | 0984 | 192 | 0681 | 273 | 0056 |
| **Oz** | 78 | 0193 | 112 | 0951 | 115 | 0853 | 91 | 0445 | 108 | 0951 | 123 | 0987 | 150 | 0938 |
| **O2** | 78 | 0193 | 110 | 1 | 126 | 0522 | 89 | 0397 | 111 | 0984 | 215 | 0454 | 246 | 0183 |
